# Supplementary material for: Quark Cheese Processed by Dense-Phase Carbon Dioxide: Shelf-Life Evaluation and Physiochemical, Rheological, Microstructural and Volatile Properties Assessment
Source: Foods. 2022 Aug 5;11(15):2340. doi: 10.3390/foods11152340 (PMC9367703; doi:10.3390/foods11152340)
Supplement: Supplementary file 1 [file foods-11-02340-s001.zip › foods-1818269-supplementary.pdf]

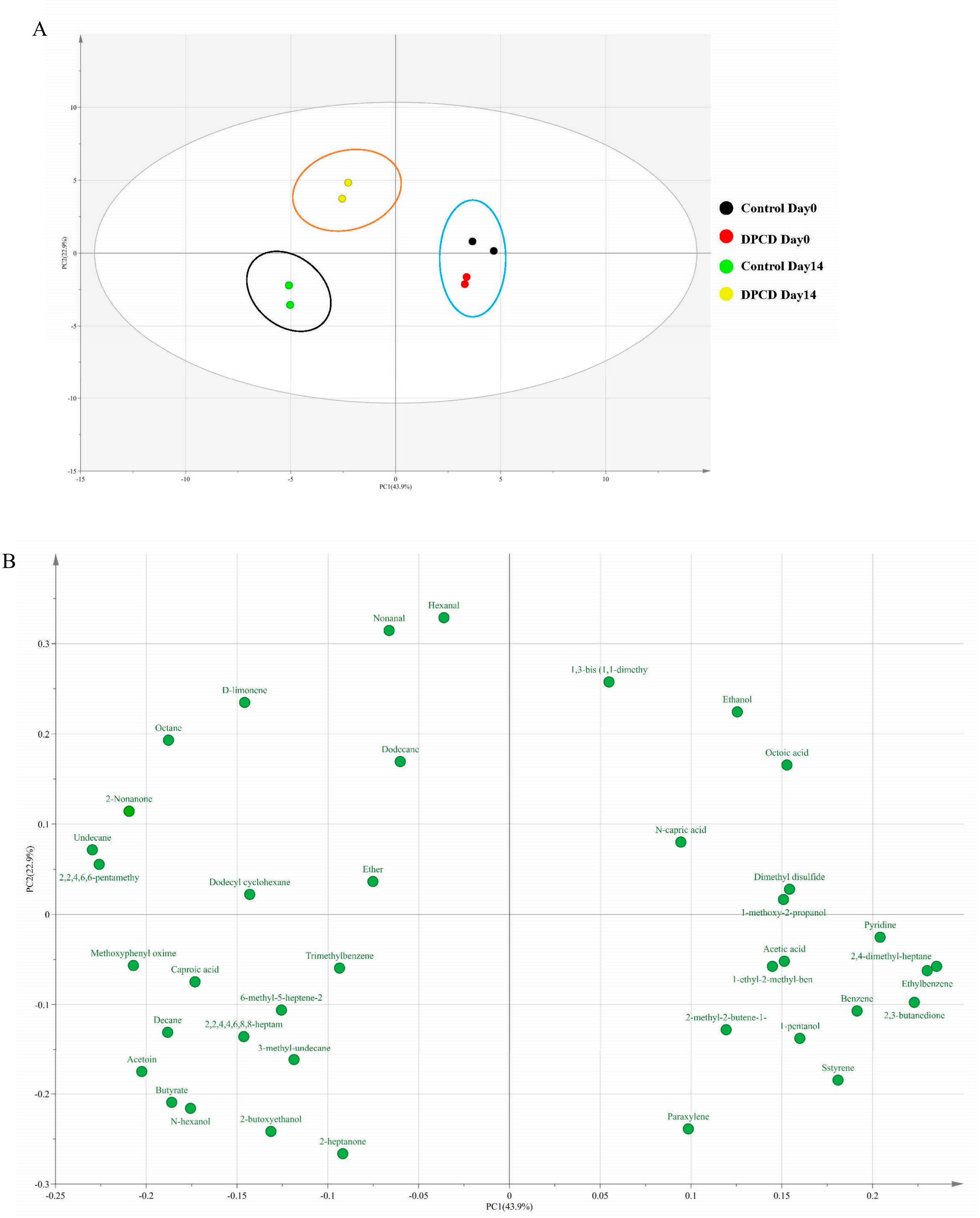
A
Control Day0
DPCD Day0
Control Day14
DPCD Day14
PC1(43.9%)
PC2(22.9%)
B
PC1(43.9%)
PC2(22.9%)
Hexanal
Nonanal
1,3-bis (1,1-dimethy
D-limonene
Ethanol
Octane
Dodecane
Octoic acid
2-Nonanone
Undecane
N-capric acid
2,2,4,6,6-pentamethy
Ether
Dodecyl cyclohexane
Dimethyl disulfide
1-methoxy-2-propanol
Pyridine
Methoxyphenyl oxime
Trimethylbenzene
Acetic acid
2,4-dimethyl-heptane
Caproic acid
1-ethyl-2-methyl-ben
Ethylbenzene
6-methyl-5-heptene-2
Benzene
Decane
2,2,4,4,6,8,8-heptam
2-methyl-2-butene-1-
2,3-butanedione
1-pentanol
3-methyl-undecane
Acetoin
Sstyrene
Butyrate
N-hexanol
2-butoxyethanol
Paraxylene
2-heptanone

C

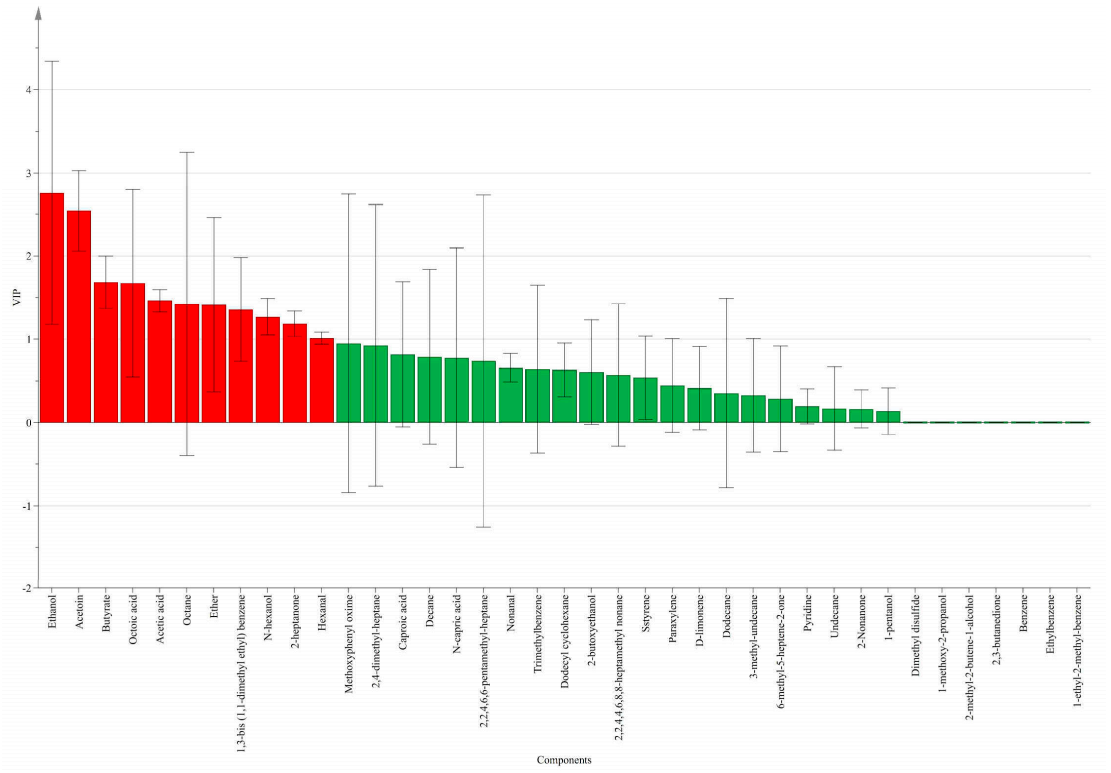

Figure S1 Non-targeted metabonomics characterization of volatile compounds from cheese by PCA and OPLS-DA. (A) PCA score plot. (B) PCA loading plot. (C) OPLS-DA VIP plot

Table S1 Power law rheological parameters (k', n, k', n', k'', n'') of control and dense phase carbon dioxide (DPCD) processed quark cheese stored at 4°C

Treatment	Storage time	K	n	K'	n'	K''	n''
Control	Day0	$158.12±29.56^{Aa}$	$-0.095±0.086^{Ba}$	$6002.80 ±1202.80^{Aa}$	$0.125 ±0.000^{Aa}$	$1527.23±271.34^{Aa}$	$0.176 ±0.000^{Aa}$
	Day14	$127.14±11.84^{a}$	$-0.021±0.019^{a}$	$4911.66 ±308.48^{a}$	$0.130 ±0.008^{a}$	$1288.60 ±57.89^{a}$	$0.179 ±0.007^{a}$
DPCD	Day0	$525.56±36.42^{Bb}$	$-0.237±0.050^{Aa}$	$16773.06 ±401.41^{Bb}$	$0.140 ±0.000^{Bb}$	$4511.54±118.25^{Bb}$	$0.174 ±0.002^{Aa}$
	Day14	$426.46±11.39^{a}$	$-0.330±0.052^{a}$	$9612.31 ±911.06^{a}$	$0.132 ±0.001^{a}$	$2538.44 ±220.01^{a}$	$0.178 ±0.007^{a}$

Control represents the native cheese without DPCD treatment.

Data are mean values ± standard deviation (n=3). The different lowercase letters indicate significant differences ($P < 0.05$) in the rheological parameters with varying storage time; the different capital letters indicate significant differences ($P < 0.05$) in the rheological parameters with or without DPCD treatment on day 0.

Table S2 Compositions and contents of volatile compounds of control and dense phase carbon dioxide (DPCD) processed cheese stored at 4℃

Number	Name	CAS	Retention time (min)	Control		DPCD	
				Day 0	Day 14	Day 0	Day 14
**Sulphocompounds**							
1	Dimethyl disulfide	000624-92-0	6.716	0.12±0.02^{A}	-	0.13±0.05^{A}	-
**Aliphatic hydrocarbons**							
2	2,4-dimethylheptane	002213-23-2	2.161	12.76±0.80^{Ab}	5.54±0.75^{a}	13.36±0.04^{Ab}	6.15±0.10^{a}
3	Octane	000111-65-9	2.274	7.37±0.78^{Aa}	10.65±1.19^{b}	8.68±0.18^{Ba}	12.09±0.00^{b}
4	2,2,4,6,6-pentamethylheptane	017302-37-3	3.484	0.73±0.02^{Aa}	3.80±0.52^{b}	1.03±0.02^{Ba}	3.23±1.02^{b}
5	Decane	000124-18-5	4.472	-	0.55±0.27	-	0.10±0.14
6	2,2,4,4,6,8,8-heptamethylnonane	004390-04-9	5.034	-	0.23±0.21	-	-
7	Undecane	001120-21-4	7.376	-	0.33±0.88	-	0.31±0.07
8	D-limonene	005989-27-5	1.049	-	0.07±0.1	-	0.19±0.04
9	Dodecane	000112-40-3	10.01	-	-	-	0.16±0.23
10	3-methylundecane	001002-43-3	9.419	-	0.07±0.10	-	-
**Heterocyclic compound**							
11	Pyridine	000110-86-1	10.497	0.99±0.63^{Aa}	0.32±0.06^{a}	1.68±0.18^{Ab}	0.33±0.01^{a}

12	Dodecyl cyclohexane	053044-27-2	15.877	$1.38±0.10^{a}$	$1.29±0.10^{a}$	-	1.57±0.06
**Ethers**							
13	Ether	000060-29-7	1.679	$32.05±0.65^{Aa}$	$33.79±3.22^{a}$	$33.61±1.36^{Aa}$	$33.81±0.34^{a}$
**Acids**							
14	Acetic acid	000064-19-7	18.268	$8.61±0.06^{Bb}$	$2.75±0.18^{a}$	$4.09±0.05^{Aa}$	$4.24±0.13^{a}$
15	Butanoic acid	000107-92-6	22.696	$3.55±0.09^{Ba}$	$5.21±0.27^{b}$	$2.55±0.04^{Aa}$	$3.23±0.29^{b}$
16	Caproic acid	000142-62-1	27.529	$9.44±0.19^{Ba}$	$9.97±0.25^{b}$	$7.08±0.03^{Aa}$	$9.47±0.34^{b}$
17	Octoic acid	000124-07-2	31.849	$7.36±0.25^{Bb}$	$4.96±0.45^{a}$	$6.61±0.33^{Aa}$	$7.07±0.63^{a}$
18	N-capric acid	000334-48-5	35.843	$1.26±0.43^{Aa}$	$0.65±0.92^{a}$	$1.06±0.03^{Aa}$	$1.06±0.40^{a}$
**Alcohols**							
19	Ethanol	000064-17-5	3.452	$1.50±0.33^{A}$	-	$8.35±0.21^{Bb}$	$5.42±1.72^{a}$
20	2-propanol, 1-methoxy -	000107-98-2	9.254	$0.14±0.03^{A}$	-	$2.84±1.52^{B}$	-
21	1-pentanol	000071-41-0	12.679	$0.42±0.12^{B}$	0.01±0.02	$0.11±0.03^{A}$	-
22	2-methyl-2-butene-1-alcohol	004675-87-0	14.76	0.09±0.01	-	-	-
23	1-hexanol	000111-27-3	15.617	$0.07±0.01^{a}$	$1.13±0.21^{b}$	-	-
24	2-butoxyethanol	000111-76-2	16.669	$0.20±0.05^{Aa}$	$0.40±0.05^{b}$	$0.15±0.01^{Aa}$	$0.12±0.18^{a}$
25	Benzyl alcohol	000100-51-6	28.494	-	0.07±0.11	-	-
**Aldehyde**							
26	Hexanal	000066-25-1	7.077	$0.09±0.01^{Aa}$	$0.07±0.09^{a}$	$0.30±0.01^{Ba}$	$0.78±0.05^{b}$
27	Nonanal			-	-	$0.04±0.00^{a}$	$0.30±0.03^{b}$

**Ketones**							
28	2,3-butanedione	000431-03-8	4.278	1.41±0.05^{B}	-	1.07±0.18^{A}	-
29	2-heptanone	000110-43-0	10.245	0.28±0.05^{Aa}	0.99±0.14^{b}	0.46±0.03^{B}	-
30	Acetoin	000513-86-0	13.696	6.66±0.38^{Ba}	11.05±0.16^{b}	4.20±0.06^{Aa}	6.52±0.10^{b}
31	6-methyl-5-heptene-2-one	000110-93-0	15.047	0.03±0.05^{Aa}	0.12±0.05^{a}	0.06±0.00^{Aa}	0.05±0.07^{a}
32	2-Nonanone	000821-55-6	14.486	0.05±0.00^{Aa}	0.12±0.03^{b}	0.06±0.00^{Ba}	0.14±0.01^{b}
**Aromatic hydrocarbon**							
33	Benzene	000071-43-2	3.526	1.23±0.38^{Aa}	0.66±0.62^{a}	-	-
34	Ethylbenzene	000100-41-4	8.163	0.06±0.01^{A}	-	0.07±0.01^{A}	-
35	Paraxylene	000108-38-3	8.572	0.26±0.05^{Aa}	0.25±0.08^{a}	0.29±0.00^{Ab}	0.09±0.13^{a}
36	1-ethyl-2-methylbenzene	000611-14-3	11.288	0.04±0.06^{A}	-	0.05±0.07^{A}	-
37	Styrene	000100-42-5	12.496	0.77±0.03^{Ab}	0.59±0.07^{a}	0.87±0.08^{Ab}	0.37±0.13^{a}
38	Mesitylene	000108-67-8	13.113	0.10±0.01^{Aa}	0.34±0.49^{a}	0.12±0.01^{Aa}	0.11±0.01^{a}
39	1,3-bis (1,1-dimethyl ethyl) - benzene	001014-60-4	17.441	0.97±0.24^{Aa}	2.55±0.38^{b}	1.06±0.10^{Aa}	2.36±0.10^{b}
**Other compounds**							
40	Methoxy phenyl oxime	1000222-86-6	34.244	-	1.35±0.85	-	0.71±0.17

Control represents the native cheese without DPCD treatment.

Data are mean values ± standard deviation (n=2). The different lowercase letters indicate significant differences ($P < 0.05$) in the content of volatile components with varying storage time; the different capital letters indicate significant differences ($P < 0.05$) in the volatile components with or without DPCD treatment on day 0.
